# Preliminary mechanistic insights of a brain-penetrant microtubule imaging PET ligand in a tau-knockout mouse model

**DOI:** 10.1186/s13550-022-00912-z

**Published:** 2022-07-26

**Authors:** Naresh Damuka, Miranda E. Orr, Avinash H. Bansode, Ivan Krizan, Mack Miller, Jillian Lee, Shannon L. Macauley, Christopher T. Whitlow, Akiva Mintz, Suzanne Craft, Kiran Kumar Solingapuram Sai

**Affiliations:** 1 Department of Radiology, Wake Forest School of Medicine, Winston Salem, NC 27157 USA; 2 Department of Gerontology, Wake Forest School of Medicine, Winston Salem, NC 27157 USA; 3 Department of Radiology, Columbia Medical Center, New York, NY 10032 USA

**Keywords:** Positron emission tomography (PET), Microtubules, Alzheimer’s disease, Biomarker, Tau imaging, And Biodistribution

## Abstract

**Background:**

Microtubules (MTs) are critical for cell structure, function, and survival. MT instability may contribute to Alzheimer’s disease (AD) pathogenesis as evidenced by persistent negative regulation (phosphorylation) of the neuronal microtubule-associated protein tau. Hyperphosphorylated tau, not bound to MTs, forms intraneuronal pathology that correlates with dementia and can be tracked using positron emission tomography (PET) imaging. The contribution of MT instability in AD remains unknown, though it may be more proximal to neuronal dysfunction than tau accumulation. Our lab reported the first brain-penetrant MT-based PET ligand, [^11^C]MPC-6827, and its PET imaging with this ligand in normal rodents and non-human primates demonstrated high brain uptake and excellent pharmacokinetics. Target engagement and mechanism of action using in vitro, in vivo, and ex vivo methods were evaluated here.

**Methods:**

In vitro cell uptake assay was performed in SH-SY5Y neuronal cells with [^11^C]MPC-6827, with various MT stabilizing and destabilizing agents. To validate the in vitro results, wild type (WT) mice (*n* = 4) treated with a brain-penetrant MT stabilizing drug (EpoD) underwent microPET/CT brain imaging with [^11^C]MPC-6827. To determine the influence of tau protein on radiotracer binding in the absence of protein accumulation, we utilized tau knockout (KO) mice. In vivo microPET imaging, ex vivo biodistribution, and autoradiography studies were performed in tau KO and WT mice (*n* = 6/group) with [^11^C]MPC-6827. Additionally, α, β, and acetylated tubulin levels in both brain samples were determined using commercially available cytoskeleton-based MT kit and capillary electrophoresis immunoblotting assays.

**Results:**

Cell uptake demonstrated higher radioactive uptake with MT destabilizing agents and lower uptake with stabilizing agents compared to untreated cells. Similarly, acute treatment with EpoD in WT mice decreased [^11^C]MPC-6827 brain uptake, assessed with microPET/CT imaging. Compared to WT mice, tau KO mice expressed significantly lower β tubulin, which contains the MPC-6827 binding domain, and modestly lower levels of acetylated α tubulin, indicative of unstable MTs. In vivo imaging revealed significantly higher [^11^C]MPC-6827 uptake in tau KOs than WT, particularly in AD-relevant brain regions known to express high levels of tau. Ex vivo post-PET biodistribution and autoradiography confirmed the in vivo results.

**Conclusions:**

Collectively, our data indicate that [^11^C]MPC-6827 uptake inversely correlates with MT stability and may better reflect the absence of tau than total tubulin levels. Given the radiotracer binding does not require the presence of aggregated tau, we hypothesize that [^11^C]MPC-6827 may be particularly useful in preclinical stages of AD prior to tau deposition. Our study provides immediate clarity on high uptake of the MT-based radiotracer in AD brains, which directly informs clinical utility in MT/tau-based PET imaging studies.

**Supplementary Information:**

The online version contains supplementary material available at 10.1186/s13550-022-00912-z.

## Background

Microtubules (MTs) are essential components of the cytoskeleton. They act as a substrate for information processing and assist in subcellular molecular trafficking and signaling across cells and tissues [1]. In brain cells, their structural integrity and stability is critical for cellular signaling, conduction, and axoplasmic transport, all of which are essential for cognitive and neurologic functions at a molecular level [2–5]. Rapid information processing necessitates highly dynamic MTs, which is achieved through polymerization of α- and β-tubulin subunits. The delicate balance between MT growth and breakdown is regulated by MT-associated proteins (MAPs) reversibly binding to and stabilizing tubulin subunits. For example, the MAP tau acquires post-translational modifications at multiple epitopes that negatively regulate its MT binding affinity i.e., phosphorylation [6–8]. Aberrant tau processing causes a group of diseases, referred to as tauopathies, which includes Alzheimer’s disease (AD) [9].

Postmortem brain tissue from patients with AD include greater amounts of twisted/unstable MT filaments than age-matched controls and high levels of phosphorylated tau that correlate with the duration and severity of AD [10]. However, it still remains unclear whether MT abnormalities have a casual and/or early role in the AD process or represent a common end point downstream of the neurodegenerative cascade. Imaging real-time molecular stability of MTs would inform whether their disruption occurs prior to, concomitant with, or after classical AD biomarkers (i.e., amyloid beta (Aβ) and tau accumulation) [11].

Positron Emission Tomography (PET)-based imaging agents provide an opportunity to monitor MT stability throughout the lifespan and in various conditions of health and disease. As Aβ and tau accumulates in the AD brain, MT stability/integrity is heavily compromised. Therefore, evaluation of a MT-tracking PET radiotracer would determine whether MT instability is an early indicator of neuronal health and brain functions prior to the onset of AD pathology and symptoms. The same tool would apply dozens of tauopathies, and non-neurological diseases where MT instability occurs.

Our lab reported the automated radiochemical synthesis of the first brain-penetrating MT PET ligand, [^11^C]MPC-6827, and its in vivo imaging utility in normal mice, rats and nonhuman primates [11, 12]. We demonstrated ideal test–retest PET imaging characteristics, high brain uptake, and excellent pharmacokinetics and specificity in these animal models. MPC-6827 is a high-affinity, selective β-tubulin binding [13] MT agent (IC_50_ = 1.5 nM) with ideal pharmacokinetics that has demonstrated suppressed tumor growth in a variety of animal models and proven safe for use in human clinical trials for the treatment of glioblastoma and other advanced cancers [13, 14]. It crosses the BBB in mice, rats, and dogs and is distributed rapidly, with approximately 14–30 times higher brain-to-plasma ratios [15]. PET radiolabeling of MPC-6827 at the methoxy site may lead to non-radioactive metabolites, but they would not interfere with the binding outcome [14, 16, 17]. Although, clinical outcomes suggest that the drug may have limited success in cancer therapy [18, 19], (a) the penetration of the BBB, (b) lack of multiple drug resistance, (c) selective on-target specific binding, and (d) safety profile in humans are the merits of [^11^C]MPC-6827 as a potential CNS PET imaging agent. The goal of this study is to provide mechanistic insight to [^11^C]MPC-6827 retention in the brain. As a first step to validate [^11^C]MPC-6827 uptake, here we demonstrated its imaging utility, including in vitro, in vivo, and ex vivo selectivity and specificity using a tau-knockout (KO) mouse model.

## Methods

### Chemicals

MPC-6827 hydrochloride (Cat. # 5231) was purchased from Tocris (Minneapolis, MN, USA) and Epothilone d (EpoD) from Cayman Chemicals (Ann Arbor, MI, USA). Tau46 mouse antibody (Cat. # 4019), 4019), β-Tubulin (Cat. # 2128), α-Tubulin (Cat. # 3873), Acetyl α-Tubulin (Cat. # 5335T), β-Tubulin (9F3) Rabbit mAb HRP Conjugate (cat # 5346) and α-Tubulin (DM1A) Mouse mAb HRP Conjugate (cat #12351), and HRP-conjugated secondary antibody (Cat. # 7076) were obtained from Cell Signaling Technology (Danvers, MA, USA). The MT/tubulin assay kit (Cat. # BK038) was obtained from Cytoskeleton, Inc. (Denver, CO, USA.) RIPA lysis buffer (Cat. # 20-188), protease inhibitor (Cat. # P8340), eosin Y (Cat. # P8340), and all the anhydrous reagents and solvents were purchased from Sigma-Aldrich (St. Louis, MO, USA). PierceTM BCA protein kit was obtained from Thermoscientific (Cat. # 23225). Modified Hematoxylin Solution (ab220365) was obtained from Abcam (Cambridge, UK). Exposer cassettes (BAS IP SR 2025) were purchased from Cytiva (Marlborough, MA, USA). Desmethyl MPC6827 (precursor for [^11^C]MPC-6827 radiochemistry) was purchased from ABX biochemical compounds (Radeberg, Germany).

### Animal sources

Female Balb/C mice (6 months old) for EpoD treatment studies were purchased from Jackson Laboratory (Bar Harbor, ME, USA). Female Mapt^0/0^ (referred to as tau KO), and wild type (WT) mice (BG.129X1-Maptt^m1Hnd^) were purchased from the Jackson Laboratory (Bar Harbor, ME, USA). All animal experiments were conducted under eIACUC approved protocols in compliance with the guidelines for the care and use of research animals established by Wake Forest Medical School Animal Studies Committee.

### Radiochemistry

[^11^C]MPC-6827 was produced following our reported procedures [11]. Briefly, desmethyl MPC-6827 (ABX) was bubbled with [^11^C]MeI in a NaOH/DMF solvent in the GE-FXC radiochemistry module at 80 °C for 5 min, followed by semi-prep HPLC column purification, and C18 SepPak elution with 10% ethanol in saline (*n* > 80 productions). Chemical, and radiochemical purity and specific activity of radioactive aliquots were determined by QC-HPLC testings. Radiochemical synthesis, including [^11^C]CO_2_/MeI transfer, reaction, HPLC purification and radiotracer formulation was completed in 50 min.

### In vitro cell uptake assays

Cell-binding assays in vitro were performed in SH-SY5Y cells with [^11^C]MPC-6827 following our published protocols [20, 21]. SH-SY5Y cells are patient-derived neuroblastoma cells routinely used to study neurotransmitter- and receptor-based effects in brain cells [22–24]. They (0.5 × 10^6^ cells/well) were treated with 1.0 µM of (a) stabilizing agents that increase MT polymerizations (paclitaxel, EpoD) [1, 25] or destabilizing agents increasing free tubulin content (vinblastine, mertasine) [26, 27] and 3 h later, [^11^C]MPC-6827 (0.074 MBq/well) was added and incubated for 30 min at RT (*n* = 6/group). To demonstrate tracer specificity [28], a subgroup of cells (*n* = 4) was pre-treated with the non-radioactive MPC-6827 (1.0 µM), radiotracer was added 60 min later, and the sample was incubated for 30 min. All the cells were washed with PBS and lysed with 1 N NaOH. Finally, the lysate from each well was γ-counted (PerkinElmer, Waltham, MA, USA) and counts-per-minute (cpm) values were normalized to the amount of radioactivity added to each well. Protein concentration was estimated in a subgroup of cells (*n* = 3) using the established Pierce BCA Protein Assay Kit from ThermoFisher [28, 29]. Cpm values were then matched to protein concentration per well, and the data expressed as %ID/mg of protein in each well.

To further investigate the tubulin selectivity of [^11^C]MPC-6827 in SH-SY5Y cells, tubulin assay was performed using the commercially available kit-based assay (Cytoskeleton Inc, USA). Briefly, SH-SY5Y cells (7 × 10^6^ cells/well) were pretreated with 1.0 µM of same MT stabilizing (EpoD, paclitaxel) and destabilizing agents (mertasine, vinblastine) for 3 h (*n* = 3/group). Radiotracer (0.074 MBq/well) was then added and incubated for 30 min at RT. Samples were loaded on to ultra-centrifuge following the kit-based assay parameters to separate stabilized/bound and destabilized/free tubulins. Stabilized tubulins from low-speed centrifugation (1000×*g* for 5 min at 37 °C) and destabilized tubulins from high speed centrifugation (100,000×*g* for 20 min at 37 °C) were collected separately and their associated radioactivity was measured using a γ counter (Perkin Elmer). Cpm values were then matched to protein concentration per well, and the data expressed as %ID/mg of protein in each well.

### MicroPET/CT imaging in tau KO and WT mice

Small animal microPET/CT imaging [11] was performed in anesthetized tau-KO and WT mice (*n* = 8/group) at 6 months of age. All mice received a bolus injection of [^11^C]MPC-6827 (18.1 ± 0.01 MBq) via the tail vein. For the microPET/CT scans, the head/brain area was centered in the field of view, and the list-mode data from the emission scans were reframed into a 0-60 min dynamic binning sequence [30]. Time-activity curves (TACs) were obtained, and radioactive uptake was expressed as standardized uptake values (SUVs) using the PMOD analysis software. Regions of interest (ROIs) were drawn for the whole brain and commonly AD-affected regions including cortex, striatum, hippocampus, and cerebellum, using Π-pmod Atlas software.

### EpoD treatment

To validate the in vitro MT-stabilization cell uptake results, normal Balb/C mice (*n* = 6, 6 mo old) were treated with epothilone D (EpoD), a brain-penetrant, MT-stabilizing drug commonly used in tauopathies. [^11^C]MPC-6827 brain PET/CT imaging was performed at baseline and after 3 days of acute treatment of EpoD treatment (3.0 mg/kg intraperitoneal injection). Whole-brain SUV_max_ was calculated and compared between the baseline and post-drug treatment scans.

### Biodistribution studies

Post-PET radiotracer tissue biodistribution studies were performed with [^11^C]MPC-6827 in the KO and WT mice (*n* = 4/group). After dynamic brain imaging from 0 to 60 min, mice were euthanized, and samples of brain, blood, heart, lung, liver, spleen, pancreas, kidney, and muscle were harvested, weighed, and γ-counted using a Wallac Gamma Counter (PerkinElmer) [11, 30] with a standard dilution of the injectate. Percentages of the injected dose per gram of tissue (%I.D/g; mean ± SD) were calculated and decay-corrected and were summarized in Table 1.

**Table 1:** Post-PET biodistribution results from wild type (WT) and tau knockout (KO) mice (*n* = 6/group) with % injected dose (ID)/mg of tissue ± standard deviation (SD) after IV injection of [^11^C]MPC-6827 (3.7 ± 0.05 MBq); **p = 0.004 for brain uptake

Organ	WT (%ID/mg ± SD)	Tau KO (%ID/mg ± SD)
Blood	0.615 ± 0.12	0.56 ± 0.05
Brain	1.03 ± 0.31	1.76 ± 0.29
Heart	0.56 ± 0.07	0.74 ± 0.03
Lung	0.77 ± 0.11	1.08 ± 0.09
Liver	3.12 ± 0.89	3.76 ± 0.77
Spleen	0.82 ± 0.21	0.68 ± 0.04
Kidneys	8.22 ± 1.4	8.51 ± 1.12
Pancreas	0.44 ± 0.01	0.32 ± 0.02
Muscle	0.14 ± 0.01	0.11 ± 0.01

### Autoradiography studies

Autoradiography studies in vitro were performed on postmortem frozen brain sections from tau KO and control mice (*n* = 6/group) following published protocols [30]. Briefly, the sagittal sections were mount on a glass slides (Super frost plus slides, Fisher Scientific, Waltham, MA), air-dried for 30 min and incubated in PBS (pH 7.4) for 10 min to remove any endogenous binding. Additionally, a sub-section of tau KO brains (*n* = 4) were self-blocked with non-radioactive MPC-6827 (10 µM) 1 h prior to radiotracer treatment. The slides were air-dried and ~ 0.5 MBq of [^11^C]MPC-6827 in PBS was added to each slide and incubated for 30 min. The slides were then washed with PBS (3×) and water (1×) at 4 °C and quickly air-dried. Slides containing the brain tissues were exposed to radioluminographic imaging cassette BAS IP SR 2025 (GE Healthcare) for 12 h at − 20 °C and scanned with a GE Amersham Typhoon scanner (25 µm pixel size). Autoradiographs were analyzed using ImageQuant TL 8.2 and ROI were manually drawn (see Additional file 1: 3), and specific binding in each ROI was calculated and expressed as photo-stimulated luminescence signals per square millimeter (PSL/mm^2^).

### Tubulin assays

To investigate differences in MT stability between tau KO and control WT mouse brains (*n* = 3/group), we performed two different assays: (a) a commercially available MT-based assay (Cytoskeleton, Inc.,) [31–33] and (b) capillary electrophoresis immunoblotting experiments.

### Cytoskeleton-based assay

This commercially available kit separates large complexes of polymerized MTs attached to nuclei and Golgi bodies into bound or non-polymerized/free tubulins. Briefly, prefrontal brain sections from euthanized mice were immediately placed in MT stabilization buffer (kit-based preparation) using a battery operated handheld homogenizer at 37 °C. Tissue lysates were centrifuged at 1000 × *g* for 5 min to separate them to nuclei and Golgi bodies (low-speed pellet), then supernatant samples were re-centrifuged at high speed 100,000 × *g* for 1 h at 37 °C to separate bound and free tubulins. The high-speed pellet was diluted in a microtubule depolymerization buffer (kit-based preparation). After the bound and free tubulin fragments were isolated, they were all slowly loaded onto a 12% SDS-polyacrylamide gel (PAGE) as recommended by the kit. The samples were then transferred onto a nitrocellulose membrane and allowed to settle for an hour at room temperature (RT) in TBST (tris-buffered saline and polysorbate 20). The membranes were incubated in primary tubulin antibody (1:2000) at 4 °C overnight, then washed with TBST (3 × 15 min) at RT with agitation. Next, they were incubated with HRP-conjugated anti-sheep secondary antibody (1: 10,000) for an additional hour and washed again in TBST (3 × 15 min) at RT. Finally, the tubulin bands were visualized using an Enhanced Chemiluminescence kit, and images were captured using a Amersham Imager 600—IA600 instrument, using the Molecular ImagJ analysis software to quantify tubulin concentrations.

### Capillary electrophoresis immunoblotting

These assays were performed using Jess™ western analyses according to the manufacturer's protocol (ProteinSimple, BioTechne, Santa Clara, CA). This technology is more sensitive and quantitative than traditional western immunoblotting, generating highly reproducible electropherograms [34–36]. In brief, lysates were diluted to 0.4 μg/µL in sample buffer, added to a master mix containing dithiothreitol (DTT) and a fluorescent molecular weight marker, then heated at 95 °C for 5 min. The chemiluminescent substrate, primary antibody, HRP-conjugated secondary antibody, NIR-conjugated secondary antibody, protein normalization reagent, blocking reagent, samples, and separation and stacking matrices were dispensed into 384-well total protein plates. Subsequent separation electrophoresis and immunodetection steps in the capillary system were fully automated. Simple western analysis was carried out at RT using instrument default settings. Primary antibodies used included rabbit anti-β-tubulin (rabbit monoclonal 9F3 [Cell Signaling Technology]; 1:50); mouse anti-α-tubulin (mouse monoclonal DM1A [Invitrogen, Rockford, IL, USA]; 1:50); rabbit anti-acetylated alpha tubulin (rabbit monoclonal [Cell Signaling Technology]; 1:50). All antibodies were diluted with milk-free antibody diluent (ProteinSimple). HRP-conjugated antibodies were used for mouse anti-α-tubulin and rabbit anti-β-tubulin, while NIR-conjugated antibody was used for acetyl-α-tubulin. Total α tubulin and acetylated-α-tubulin were superplexed in the same well. The digital image was analyzed with Compass software (ProteinSimple) and the protein densitometry was calculated by dividing the area under the curve of each protein of interest by the area under the curve of total protein within the capillary.

### Statistical analysis

All statistical analyses for cell uptake, microPET imaging, biodistribution, and autoradiography data were performed using GraphPad prism (version 7.0) and reported as the average value ± standard deviation. Statistical analysis was performed using a two-tailed student’s *t* test, with *p ≤ 0.05 and *α* = 0.05 being considered significant.

## Results

### Radiochemistry

[^11^C]MPC-6827 radiochemistry was fully automated and optimized in GE FXC module (see Additional file 1: 1) with a ~ 40% yield (n > 80 productions), and high radiochemical purity (> 98%), and specific activity (~ 114 ± 10 GBq/µmol); decay was corrected to the end of synthesis (EOS).

### In vitro assays

**Fig. 1:**
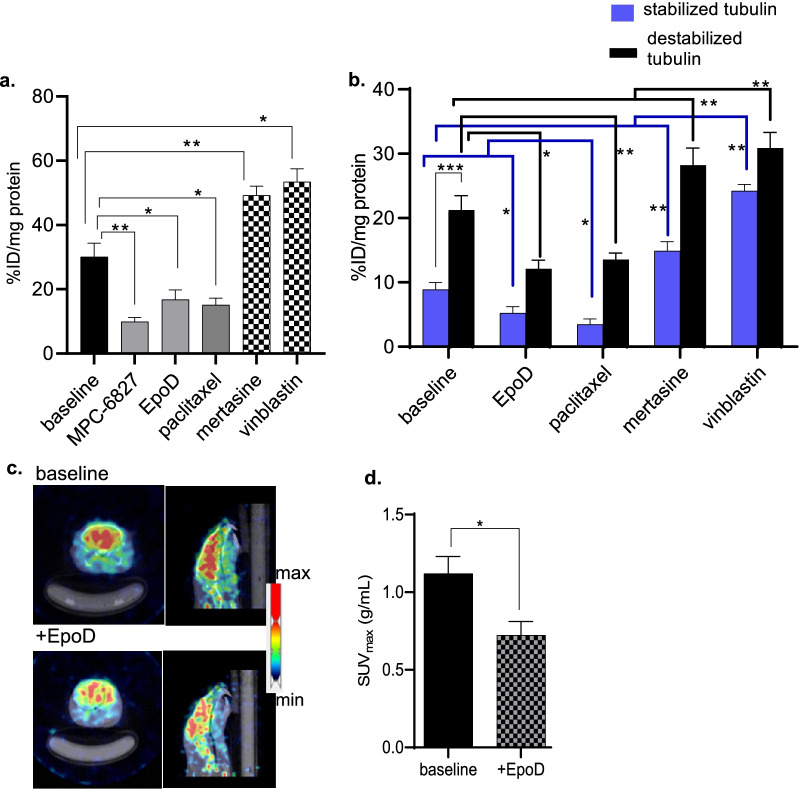
[^11^C]MPC-6827 **a** standard cell uptake **b** tubulin kit-based assay in vitro with different MT stabilizing and destabilizing agents after 30 min incubation (*n* = 6/group), using in SH-SY5Y cells; *p ≤ 0.05, **p = 0.004, ***p = 0.00014; **c** representative axial and sagittal PET/CT brain images from FVB/B6 wild type mice (*n* = 4) at baseline and after EpoD treatment with **d** their whole-brain SUV_max_ after IV injection of [^11^C]MPC-6827 (3.7 ± 0.05 MBq); *p ≤ 0.05, **p = 0.0061

Initial [^11^C]MPC-6827 radioactive uptake experiments were conducted in SH-SY5Y cells, a human neuroblastoma cell line. Cell uptake assays demonstrated differential uptake dependent upon the class of MT agents used (Fig. 1a). The MT stabilizing agents, EpoD and paclitaxel decreased radioactive uptake by ~ 44 (± 2)% and ~ 49 (± 3)%, respectively (*p = 0.036). In contrast, MT destabilizing agents mertasine and vinblastine increased uptake by 63 (± 2)% (*p = 0.046) and ~ 78 (± 2)% (*p = 0.006), respectively. In self-blocking assays, radioactive uptake was ~ 70 (± 1)% (**p = 0.0011) lower after non-radioactive MPC-6827 was added. Cytoskeleton kit-based tubulin assay with [^11^C]MPC-6827 showed > 90 (± 4)% (***p = 0.00014) increased radioactivity in destabilized/free tubulin fractions compared to the bound fractions (Fig. 1b). Also, MT stabilizers, EpoD and paclitaxel treatments decreased radioactivity by ~ 44 (± 1)% and ~ 36.8 (± 3)% (*p = 0.04) in free tubulin fractions and by ~ 62 (± 6)% and ~ 60 (± 2)% (*p = 0.05) in bound tubulin fractions, respectively, compared to the untreated fractions. While, MT destabilizers mertasine and vinblastine increased radioactivity by ~ 39 (± 3)% and ~ 43 (± 1)% (**p = 0.004) in free tubulin fractions and by ~ 66 (± 2)% and 84 (± 4)% in bound fractions, respectively, compared to untreated fractions (Fig. 1a, b). Collectively, the in vitro experiments indicated higher radiotracer uptake in conditions of MT instability.

### In vivo tests

**Fig. 2:**
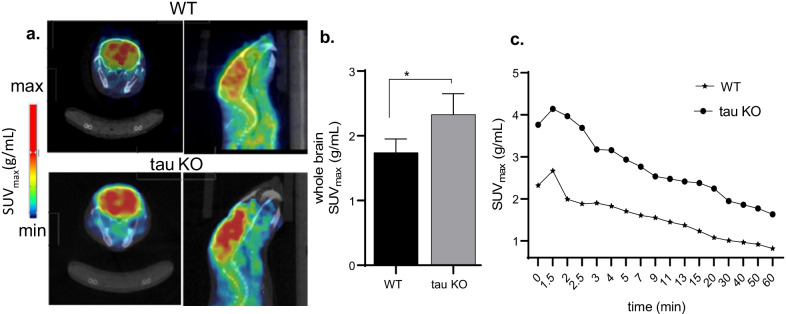
Representative 0–60 min **a** axial and sagittal PET/CT brain images from wild type (WT) and tau knock out (KO) mice (*n* = 8/group) with their whole-brain, **b** SUV_max_ and **c** TACs after IV injection of [^11^C]MPC-6827 (3.7 ± 0.05 MBq); *p = 0.032

To determine translatability of our in vitro findings, we performed complementary experiments in vivo. We focused on the blood brain barrier (BBB)-penetrant MT stabilizing agent, EpoD, that is well-published in the tauopathy literature [37–39]. Consistent with the in vitro data, in vivo microPET imaging in female Balb/C (*n* = 6) also demonstrated ~ 23 (± 1) % lower brain uptake with EpoD treatment compared to baseline scans (Fig. 1c, d, *p = 0.036). Next to determine whether our radiotracer provided uptake differences with a genetic model of aberrant neuronal microtubule stability in the absence of tau pathology, including dysregulated axons, dendrites, and network functions [40], we performed microPET scans in tau KO mice and compared them to age-matched WT mice. Western blot assay confirmed tau protein expression between genotypes (see Additional file 1: 2). MicroPET/CT imaging in tau KO and WT mice [11] showed brain penetration and high retention of [^11^C]MPC-6827 (Fig. 2a). However, SUV_max_ for whole-brain in tau KO was 33 (± 2)% higher than that measured in WT controls (Fig. 2b, 1.74 vs. 2.33; *p = 0.032). TACs also demonstrated higher initial uptake in tau KO than in age-matched controls (Fig. 2c). Both genotypes demonstrated similar radioactivity profiles, peaking at around 4 min and gradually washing out by 60 min.

### Ex vivo studies

Post-PET biodistribution results showed ~ 41% higher brain uptake in tau KO mice (%ID/g = 1.76 ± 0.31) compared to the WT controls (%ID/g = 1.03 ± 0.29, Table 1). More specifically, uptake in the cortical regions of tau KO brains was 37% higher than in controls (%ID/g = 1.11 ± 0.03 vs. 0.69 ± 0.07) with **p = 0.004.

### Autoradiography experiments

**Fig. 3:**
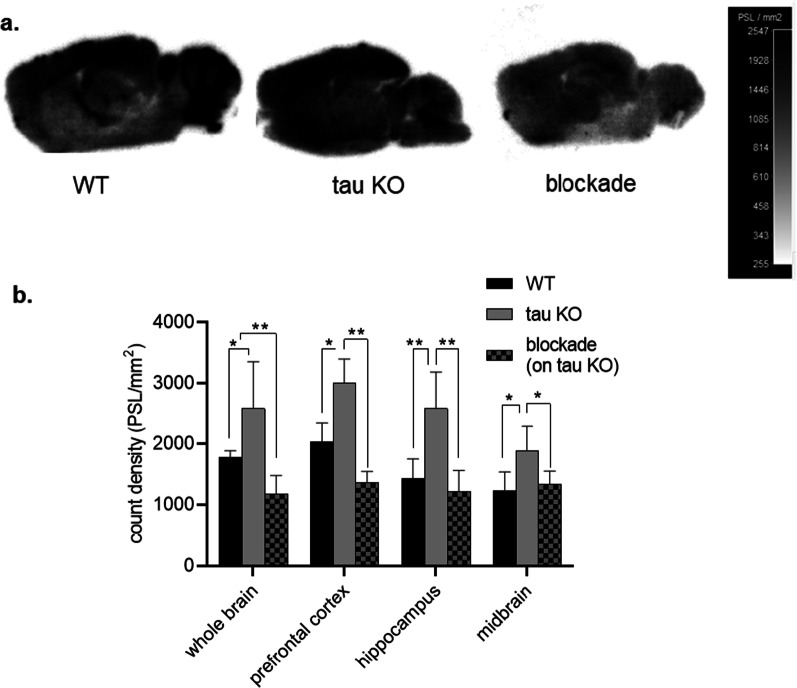
Representative **a** autoradiograms in vitro and its **b** regional quantification of [^11^C]MPC-6827 from wild type (WT), tau KO, and blockade on tau KO mouse brain tissues (*n* = 3/group); *p ≤ 0.05, **p ≤ 0.005

Additionally autoradiography experiments in vitro showed significantly higher radioactivity in the whole brain (~ 46[± 4]%, **p = 0.0028), cortex (~ 38[± 2]%, *p = 0.04), hippocampus (~ 33[± 3]%, *p = 0.035), and midbrain regions (30[± 3]%, *p = 0.015) of tau KO brains compared to age-matched controls (Fig. 3). Blocking with MPC-6827 showed ~ 63–70% (**p = 0.0014) lower radiotracer uptake in whole brain, cortex, hippocampus, and mid-brain regions compared to the untreated tau KO samples.

### Tubulin assays

**Fig. 4:**
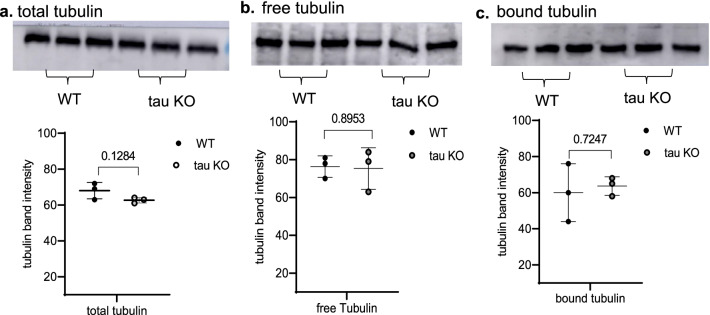
Tubulin assay using the commercially available, cytoskeleton-based MT kit with western blots of **a** total, **b** free, and **c** bound tubulins and their respective intensities in wild type (WT) and tau knock out (KO) mice (*n* = 3/group). No significant differences were observed in total, free, and bound tubulin levels

**Fig. 5:**
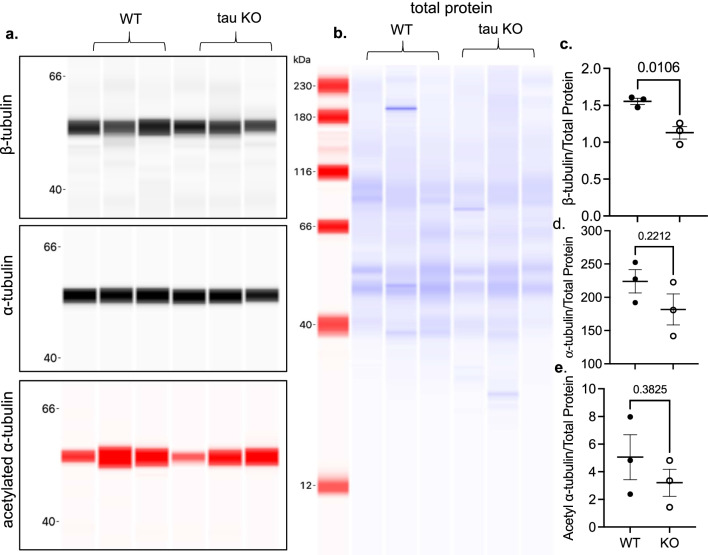
Artificial immunoblots generated from capillary electrophoresis (Jess™) using 1.2 μg protein lysate from total tubulin extracts. **a** Lysates were immunoprobed for α-tubulin, β-tubulin and acetylated α-tubulin and normalized to **b** total protein., **c**–**e** Normalized protein levels were plotted and statistically analyzed (*n* = 3/group); *p* values from unpaired *t *tests are indicated on the blots

To investigate the role of tubulin stability and subunit expression on differential brain uptake of [^11^C]MPC-6827 between the two genotypes, brain tissues from both 6 month-old tau KO and WT mice (*n* = 3/group) were analyzed for total, bound or free tubulin using a cytoskeleton-based MT kit assay where protein expression was analyzed using traditional Western blotting (Fig. 4) and tubulin subunit expression was further evaluated using capillary electrophoresis (Fig. 5). An antibody that detects global tubulin protein expression (i.e., both α- and β-tubulin) indicated a modest, non-significant decrease in tau KO mice compared to WT mice; however, free and bound tubulin expression were the same between genotypes. Capillary electrophoresis of the total tubulin fraction using antibodies specific to α- and β-tubulin subunits revealed a significant decrease in β tubulin expression in the tau KO mice compared to controls (26% reduction, *p* = 0.0106, Fig. 5a–c). The α and acetylated-α-tubulin expression were statistically similar between the two genotypes, though both were modestly lower in tau KO mice (18% and 36.7%, respectively, Fig. 5d, e). These results demonstrate that in the absence of tau protein associated MT stability (i.e., tau KO), tubulin expression patterns significantly differ than in the presence of physiological tau expression.

Collectively all in vitro cell uptake, in vivo microPET/CT imaging, and ex vivo biodistribution studies, and autoradiography (*n* = 6/group) detected a power of > 80%, a standard deviation of 0.54 units, and *α* = 0.05, with *p ≤ 0.05 indicating statistical significance.

## Discussion

We reported the automated radiochemical synthesis of the first brain-penetrating MT PET ligand, [^11^C]MPC-6827 and its in vivo PET imaging in rodents and nonhuman primates [11, 12], including high brain uptake, and excellent pharmacokinetics and specificity. Lindberg *et.al.* recently reported differential uptake in postmortem human AD brains compared to age-matched controls using autoradiography [41]. The current evidence strongly supports its clinical utility; however the implications of [^11^C]MPC-6827 uptake in normal and disease conditions are poorly understood. For example, MPC-6827 is a MT destabilizing agent that selectively binds to β tubulin suggesting higher [^11^C]MPC-6827 uptake may occur in the presence of elevated β-tubulin; however, higher radiotracer uptake in postmortem AD brain is not consistent with this mechanism of action. Using our multiple in vitro, in vivo, and ex vivo methods here, we find that higher uptake reflects unstable MTs.

The use of MT stabilizing and destabilizing agents in vitro and in vivo consistently indicated higher uptake of the radiotracer in the presence of unstable MTs (Figs. 1, 2). To evaluate the clinical significance, we chose to evaluate tau KO mice. The co-occurrence of phosphorylated tau and its accumulation among neurodegenerative diseases implies that neuronal dysfunction may involve MT instability, i.e., tau phosphorylation negatively regulates its MT-stabilizing function [42, 43]. However, the contribution of tau loss-of-function (i.e., destabilized MTs) compared to its toxic gain-of-function (e.g., tau accumulation and deposition) remains unclear. The use of tau KO mice allowed us to evaluate radiotracer uptake in association with tau dysfunction in the absence of tau deposition. In other words, if [^11^C]MPC-6827 is mechanically associated with tau loss-of-function and MT instability, we hypothesized that we would observe higher uptake in tau KO than WT mice. However, if uptake instead depends on the presence of phosphorylated tau, we anticipated higher uptake in WT than tau KO mice. Our results supported the former hypothesis. MicroPET imaging with [^11^C]MPC-6827 demonstrated higher in vivo radioactive uptake in the tau KO brains than in age-matched control WTs (Fig. 2). Ex vivo post-PET biodistribution studies also demonstrated higher brain uptake in tau KOs compared to controls (Table 1). These data suggest that higher uptake in postmortem human AD brains might reflect unstable MTs.

While in vivo microPET and ex vivo biodistribution studies both demonstrated higher radiotracer uptake in tau KO than age-matched WT mouse brains, no significant differences were detected in bound and free tubulin expression between the genotypes.

Increased radioactive uptake in tau KOs than in WT controls and decreased uptake in WT mice after treatment with an MT stabilizer (EpoD) suggest that [^11^C]MPC-6827 binding reflects unstable MTs. While the binding properties of tau in AD brains are not yet completely understood, nuclear magnetic resonance (NMR) and electron paramagnetic resonance (EPR) competitive modeling studies [44] showed some correlation between tubulin binding in the TauF4 and T2R (tau fragments) and vinblastine and colchicine binding sites. Tau stabilizes MTs and promotes a straight protocol filament conformation by binding to a hydrophobic pocket between tubulin heterodimers, which is completely inhibited by vinblastine [45]. In our data here, SH-SY5Y in vitro uptake studies showed increased [^11^C]MPC-6827 uptake when the MT destabilizer, vinblastine, was added. We also overlaid a PET/CT image atlas map acquired from a tau KO mouse (using PMOD 4.0 mouse atlas) [46] with a *Mapt* (microtubule associated protein tau) mouse in situ hybridization atlas image (Allen Mouse Brain Atlas) to visualize the correlation between high radiotracer uptake and tau-expressing brain regions (see Additional file 1: 3). The overlaid images illustrated that high concentrations of [^11^C]MPC-6827 occur in brain regions with physiologically high *Mapt* expression: hippocampus, midbrain, and cortical regions.

The results and hypothesis presented here support our preliminary PET imaging findings of high [^11^C]MPC-6827 uptake in nonhuman primates with low CSF Aβ_42_ that we previously shown to be associated with higher brain Aβ deposition [47], and therefore potentially more destabilized MTs, versus nonhuman primates with high CSF Aβ_42_ (and presumably low brain Aβ deposition, and healthy MTs) in vivo [48]. The same finding has been independently replicated in human brain (post-mortem) and rodents by other groups [41, 49]. Notably, increased [^11^C]MPC-6827 autoradiography binding in postmortem human AD brain compared to controls [41] is consistent with the results in tau KO mice here. Given that tau KO mice do not accumulate Aβ plaque or tau deposition, neuronal loss, neuroinflammation or other pathological features of AD, our data indicate that radiotracer uptake does not reflect these pathologies, but instead is altered by MT stability. Moreover, tau KO mice express significantly lower β tubulin than WT mice and modest reductions in acetylated- and α-tubulin levels indicating that the radiotracer does not bind non-specifically to total tubulin. Combined with the mechanistic studies using MT stabilizing/destabilizing agents, our results collectively indicate that [^11^C]MPC-6827 uptake reflects MT stability. A limitation of our study included the exclusive use of female mice.

As tau is a microtubule-associated protein (MAP) whose dysregulation is heavily affected in AD and related dementia, several tau-based radiotracers are currently being evaluated as potential imaging biomarkers to study neurodegeneration pathways [50–54]. Of note, these radiotracers are designed to target heavily phosphorylated tau/aggregated tau that occurs in later stages of the disease. While the growing interest in tau PET imaging will contribute to significant advancements in the field, with respect to both diagnosis and tauopathies; new radiotracers tracking the MT instability are needed to understand the early changes associated with neuronal dysfunctions. Based on our data, we hypothesize that [^11^C]MPC-6827 uptake is primarily associated with MT instability and relates to tau loss-of-function, not heavily phosphorylated/aggregated tau. Therefore, the radiotracer holds the potential for inclusion as a biomarker of neurodegeneration, to be used in parallel or alone with tau measurements to achieve an improved understanding of tauopathies.

## Conclusions

Our preliminary in vitro studies with [^11^C]MPC-6827 in SH-SY5Y cells using MT stabilizing and destabilizing agents and in vivo and ex vivo PET imaging studies of tau KO and WT mice demonstrated significant differential uptake: high radiotracer uptake with destabilizing MT agents compared to untreated baseline and high brain uptake in tau KO mice compared to controls. However, we found no significant difference in the expression of either bound and free tubulins between the tau KOs and WT controls. These data suggest that [^11^C]MPC-6827 uptake is not driven by available tubulin expression, but better reflects MT stability. We are conducting rigorous [^11^C]MPC-6827 analyses to determine metabolic plasma-blood parameters, equilibrium and association/dissociation constants and correlations between other MAPs and tau overexpression models. They will provide more mechanistic details in support of using [^11^C]MPC-6827 to image MT stability in AD and other related diseases.

## Supplementary Information

**Additional file 1.** Detailed information of the radiolabeling procedure as well as Tau immunoblotting, PET/CT overlaid on Mapt atlas as supplementary figures.

## Data Availability

All data generated or analyzed during this study are included in this published article.
