# Supplementary material for: Preliminary mechanistic insights of a brain-penetrant microtubule imaging PET ligand in a tau-knockout mouse model
Source: EJNMMI Res. 2022 Jul 26;12:41. doi: 10.1186/s13550-022-00912-z (PMC9325934; doi:10.1186/s13550-022-00912-z)
Supplement: Supplementary file 1 — Additional file 1. Detailed information of the radiolabeling procedure as well as Tau immunoblotting, PET/CT overlaid on Mapt atlas as supplementary figures. [file 13550_2022_912_MOESM1_ESM.docx]

**Preliminary mechanistic insights of a brain-penetrant microtubule imaging PET ligand in a tau-knockout mouse model**

Naresh Damuka,^#1^ Miranda E Orr,^#2^ Avinash H Bansode,^1^ Ivan Krizan,^1^ Mack Miller,^1^ Jillian Lee,^2^ Shannon L. Macauley,^2^ Christopher T Whitlow,^1^ Akiva Mintz,^3^ Suzanne Craft,^2^ Kiran Kumar Solingapuram Sai*^1^

^#^Contributed equally

*****Corresponding author

^1^Department of Radiology, Wake Forest School of Medicine, Winston Salem, NC, USA 27157; ^2^Department of Gerontology, Wake Forest School of Medicine, Winston Salem, NC, USA 27157; ^3^Department of Radiology, Columbia Medical Center, New York, NY, USA 10032

**Supplement 1: [^11^C]MPC-6827 radiochemistry**

Radiochemistry of [^11^C]MPC-6827 was performed following our reported method.^1^ Briefly, [^11^C]MeI was bubbled into the reaction vial containing 1 mg of desmethyl MPC-6827 (ABX Inc) in 0.5 mL DMF and 8-10 µL 5N NaOH in the GE FxC module. After the saturation of radioactivity, the reaction vial was heated for 5 min at 80°C and quenched with ~0.7-0.8 mL of semi-prep HPLC isocratic mobile phase (20% 0.1M aqueous ammonium formate buffer pH 6.5: 30% ACN). The aqueous reaction mixture was injected onto a C18 Phenomenex C18 semi-prep HPLC column (250 x 10mm, 10 µ), UV @ 254 nm, and a flow rate of 7 mL/min. The desired [^11^C]MPC-6827 was collected at 7.5-8.5 min into a round bottom preloaded with 55 mL of sterile water, and passed via an activated WATERS C18 SepPak cartridge (WAT036800) to trap [^11^C]MPC-6827. The radioactive product was eluted from the cartridge with 10% ethanol in saline (1 mL ethanol and 10 mL of sterile saline) into a sterile pre-weighed vial through a sterile 0.22 µ filter.

Quality control of [^11^C]MPC-6827 was performed using a WATERS analytical HPLC isocratic system with a C18 prodigy (250 x4.6 mm, 5 µm ODS-3 100° A) column at a flow rate of 1 mL/min, mobile phase of 60 % acetonitrile in 0.1 M aqueous ammonium formate solution (pH 6.5) and UV @ 254 nm. At these conditions, the radiotracer showed a single peak (>98% chemical and radiochemical purity) with a retention time of ~6.0-6.5 min. Further, the purity was authenticated with nonradioactive MPC-6827 co-injection, that demonstrated similar retention time (**Fig. 1**).

**
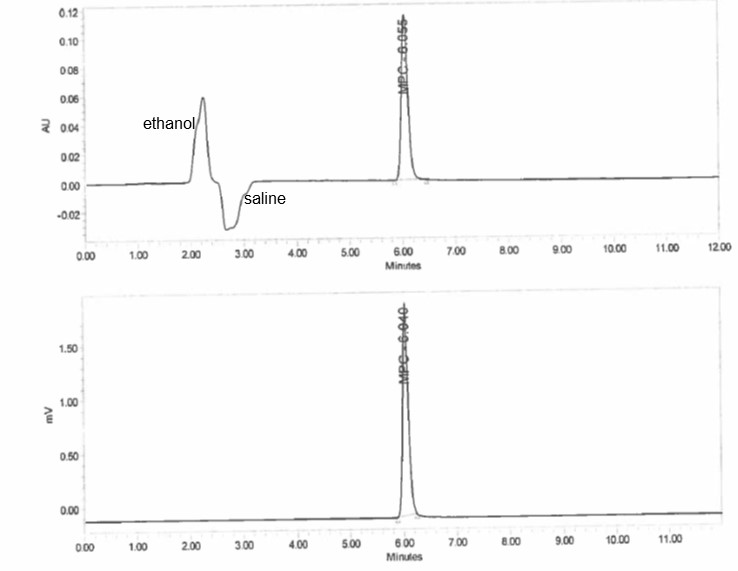
**

**Figure 1**. QC HPLC chromatogram of [^11^C]MPC-6827 co-injection with nonradioactive MPC-6827 (1 µM) demonstrating UV (upper) and radiodetector (lower) windows at a retention time of ~6.05 (+0.1) min.

**Supplement 2: Tau immunoblotting**

We routinely confirm the absence of tau expression in the tau KO mice through tau immunoblotting. The tau_46_ monoclonal antibody recognizes C-terminal amino acids 404-441, thus detecting all tau isoforms. It also cross-reacts with microtubule-associated proteins (MAPs) at 280kDa. Tau_46_ mouse mAb is routinely used^2^ to predict various isoforms of tau based on amino acid sequence. Western blot analysis was performed in WT and tau KO mouse brains (n=6/group) using the commercially available tau_46_ kit-based monoclonal antibody (mAb) assay (Cell Signaling Technology). Briefly, tau KO and WT mice (n=8/group) were euthanized and immediately decapitated. Prefrontal brain regions were carefully isolated and immersed in RIPA lysis buffer, followed by homogenization and centrifugation of the tissue at 12,000 rpm for 30 min. The supernatant was collected and used as the SDS-PAGE assay source, using Tau_46_ as the primary and HRO-conjugated anti-sheep as the secondary antibodies. Western blot^3^ signals were analyzed using ImageJ quantification software (**Fig. 2**).

WT

tau KO


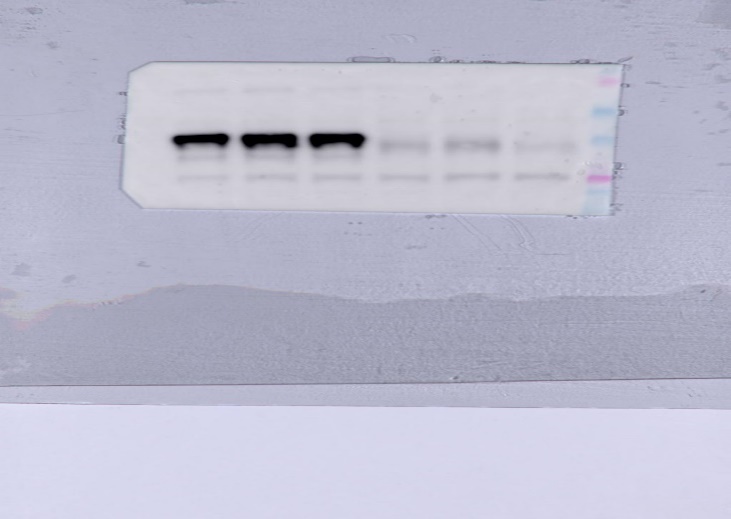


tau_46_


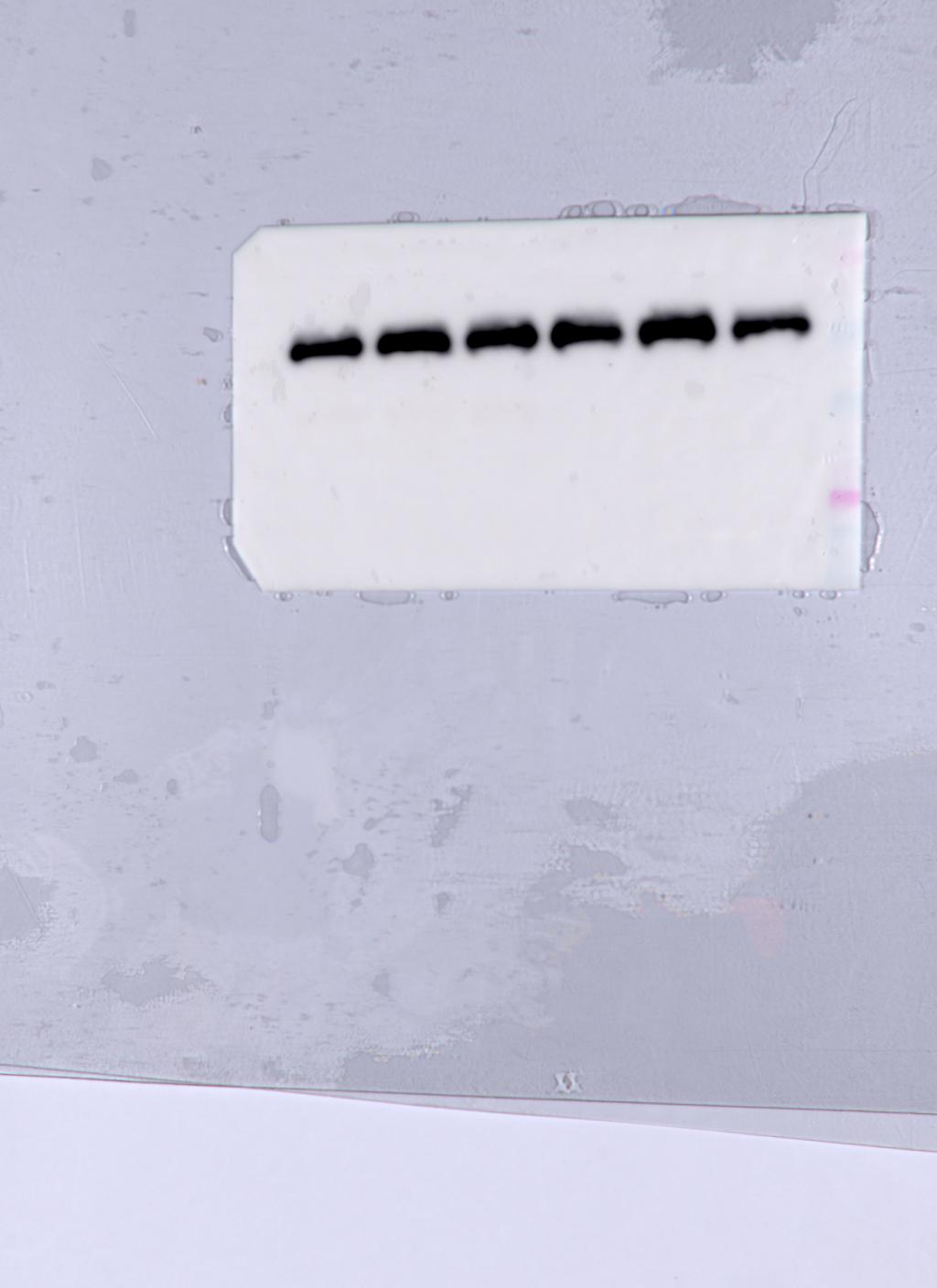


GAPDH

**Figure 2**. Representative tau_46_ and GAPDH western blots and their intensities in wild type (WT) and tau KO mice (n=8); ****p*=0.0007

**Supplement 3: PET/CT overlaid on *Mapt* atlas**

We overlaid a PET/CT image atlas map acquired from a tau KO mouse (using PMOD 4.0 mouse atlas)^4^ with a *Mapt* (microtubule-associated protein tau) mouse *in situ* hybridization atlas image (Allen Mouse Brain Atlas; <https://mouse.brain-map.org/experiment/siv?id=79556696>) to visualize the correlation between high radiotracer and tau-expressing brain regions (**Fig. 3 and Fig. 4**). The overlaid images illustrated that high concentrations of [^11^C]MPC-6827 occur in brain regions with physiologically high *Mapt* expression: hippocampus, midbrain, thalamus, and cortical regions.


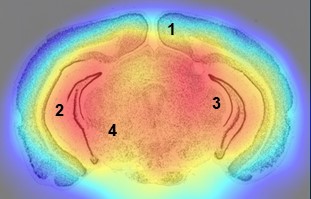


**Figure 3**. Representative PMOD-derived axial view of microPET image from a tau KO mouse overlaid on *Mapt* expression profile from Allen Mouse Brain Atlas, demonstrating high radiotracer concentrations in **1**. cortex, **2**. hippocampus, **3**. thalamus, and **4**. midbrain regions


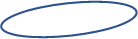

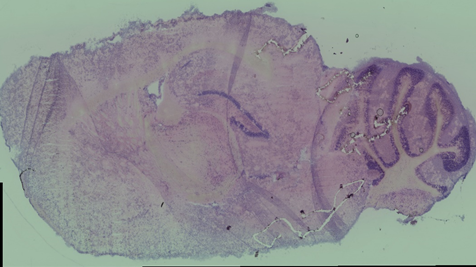

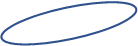

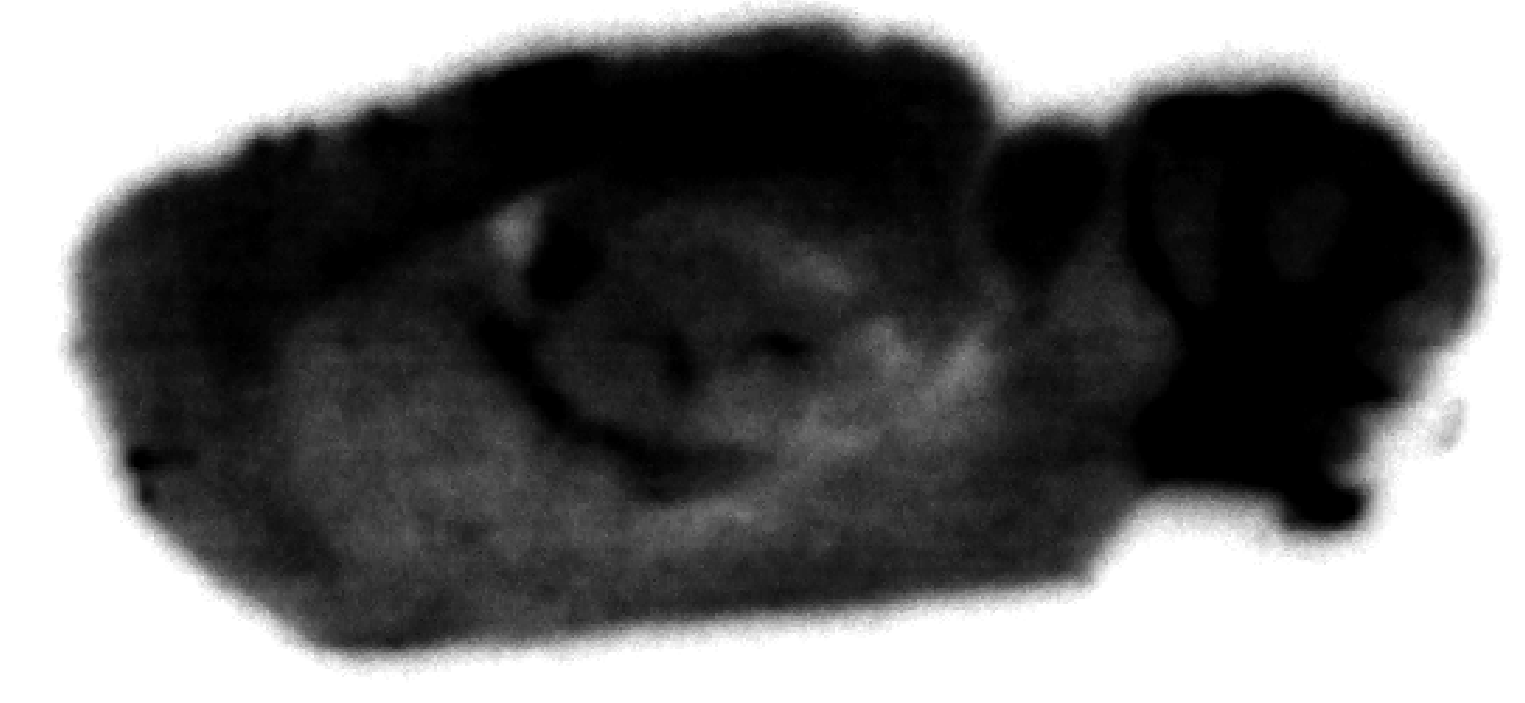


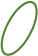

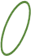

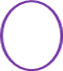

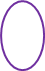


**B**. hematoxylin staining

1. [^11^C]MPC-6827

**Figure 4**. **A**. Representative autoradiograms and ROIs of [^11^C]MPC-6827 binding in a normal wild-type mouse brain sagittal sections. **B**. The ROIs were plotted based on anatomic structure of hematoxylin staining from the same mouse brain, with the following color segmentation: blue: prefrontal cortex, green: midbrain, and violet: hippocampus.

References

1. Kumar, J. S. D.; Solingapuram Sai, K. K.; Prabhakaran, J.; Oufkir, H. R.; Ramanathan, G.; Whitlow, C. T.; Dileep, H.; Mintz, A.; Mann, J. J., Radiosynthesis and in Vivo Evaluation of [11C]MPC-6827, the First Brain Penetrant Microtubule PET Ligand. *Journal of Medicinal Chemistry* **2018,** *61* (5), 2118-2123.

2. Johnson, G. V.; Stoothoff, W. H., Tau phosphorylation in neuronal cell function and dysfunction. *J Cell Sci* **2004,** *117* (Pt 24), 5721-9.

3. Naresh, D.; Bharne, D.; Saikia, P.; Vindal, V. In *Anthraquinone rich Cassia fistula pod extract induces IFIT1, antiviral protein*, 2018.

4. Hjornevik, T.; Leergaard, T.; Darine, D.; Moldestad, O.; Dale, A.; Willoch, F.; Bjaalie, J., Three-dimensional atlas system for mouse and rat brain imaging data. *Frontiers in Neuroinformatics* **2007,** *1* (4).
